# Chinese red yeast rice (*Monascus purpureus*-fermented rice) promotes bone formation

**DOI:** 10.1186/1749-8546-3-4

**Published:** 2008-03-29

**Authors:** Ricky WK Wong, Bakr Rabie

**Affiliations:** 1 Biomedical and Tissue Engineering Research Group, University of Hong Kong, Sai Ying Pun, Hong Kong SAR, China

## Abstract

**Background:**

Statin can induce the gene expression of bone morphogenetic protein-2. Red yeast rice (RYR, *Hongqu*), *i.e*. rice fermented with *Monascus purpureus*, contains a natural form of statin. This study demonstrates the effects of RYR extract on bone formation.

**Methods:**

Bone defects were created in the parietal bones of two New Zealand white rabbits. In the test animal, two defects were grafted with collagen matrix mixed with RYR extract. In the control animal, two defects were grafted with collagen matrix alone. UMR 106 cell line was used to test RYR extract *in vitro*. In the control group, cells were cultured for three durations (24 hours, 48 hours and 72 hours) without any intervention. In the RYR group, cells were cultured for the same durations with various concentrations of RYR extract (0.001 g/ml, 0.005 g/ml and 0.01 g/ml). Bicinchoninic acid (BCA) assay, 3-(4,5-dimethylthiazol-2-yl)-2,5-diphenyltetrazolium bromide (MTT) assay and alkaline phosphatase (ALP) assay were performed to measure total protein, mitochondrial activity and bone cell formation respectively.

**Results:**

The test animal showed more formation of new bone in the defects than the control animal. RYR significantly increased the optical density in the MTT assay and ALP activity *in vitro*.

**Conclusion:**

RYR extract stimulated new bone formation in bone defects *in vivo *and increased bone cell formation *in vitro*.

## Background

For treating osteoporosis or healing bone defects after trauma or surgery, it is necessary to find better medicines to enhance bone formation. Bone morphogenetic proteins (BMPs) are important regulators in osteogenic differentiation during fracture repair [1]. Wang *et al*. [2] demonstrated that bone morphogenetic protein-2 (BMP-2) caused commitment and differentiation of multi-potential stem cell line into osteoblast-like cells. In an attempt to discover small molecules that induce BMP-2 gene expression, Mundy *et al*. [3] tested more than 30 000 compounds and identified that statin was the only compound that specifically increased BMP-2 gene expression. Statin is known as a cholesterol-lowering agent that inhibits hepatic hydroxymethylglutaryl coenzyme A (HMG-CoA) reductase, the rate-limiting enzyme in the mevalonate pathway. Our previous study showed that statin increased local bone formation by 308% in 14 days in an animal model [4]. Another study indicated that the statin-mediated activation of BMP-2 promoter was a result of its ability to inhibit HMG-CoA [5]. These studies suggest that HMG-CoA reductase inhibitors may induce BMP-2 gene expression thereby increasing bone formation.

**Figure 1:**
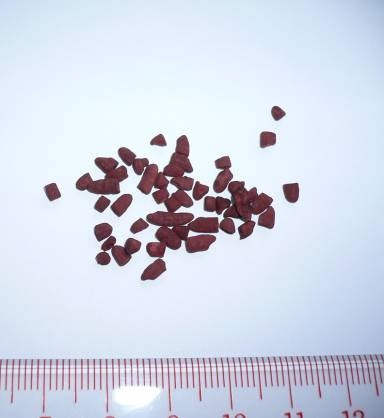
Red yeast rice (RYR, *Hongqu*).

Red yeast rice (RYR, *Hongqu*), a Chinese dietary seasoning prepared by fermenting rice with *Monascus purpureus *[6] (Figure 1), has been used in Chinese medicine for centuries. RYR contains monacolins which are a family of HMG-CoA reductase inhibitors [7]. Moreover, monacolin K is equivalent to the statin known as mevinolin or lovastatin. Other active ingredients in RYR include sterols (β-sitosterol, campesterol, stigmasterol, sapogenin), isoflavones and monounsaturated fatty acids [8]. RYR extract may promote bone formation but has not been experimentally demonstrated. The present study investigates the effects of RYR extract on bone formation *in vivo *and *in vitro*.

## Methods

### Preparation of RYR extract

RYR was purchased from a local Chinese medicine store and was identified morphologically and histologically according to standard Chinese herbal identification procedures [9]. A voucher specimen including identification and classification of the plant was preserved in the Hard Tissue Laboratory, University of Hong Kong. RYR extract was prepared according to the protocol for injection preparation of traditional Chinese medicine [10]. For every 4 g of RYR powder, 40 ml of HPLC grade water was added and the mixture was boiled with stirring on a hot plate for 4 hours. HPLC grade water was added occasionally to prevent the mixture from drying. After boiling, the final volume of the mixture was made up to 4 ml by adding HPLC grade water. The mixture was cooled to room temperature and then centrifuged (4648 × *g*). The supernatant was collected and filtered with a 0.22 μm sterile syringe filter (25JP020AS, MPS, USA) into a sterile glass bottle. Each ml of the supernatant contained 1 g of RYR extract.

### In vivo qualitative study

The method and animal model used in the present study have been previously described [4]. Four 10 × 5 mm^2 ^full-thickness bone defects were created in the parietal bones of two inbred New Zealand white rabbits. The rabbits were five months old (adult stage) and weighed between 3.5 kg to 4.0 kg. One of them was used as control while the other was used as test animal. The animal handling and experimental protocol was approved by the Committee on the Use of Live Animals in Teaching and Research, University of Hong Kong. Two defects of the test animal were grafted with collagen matrix with RYR extract; two defects of the control animal were grafted with collagen matrix alone. The animals were pre-medicated one hour before surgery with oxytetracycline hydrochloride (200 mg/ml, 30 mg/kg body weight, Tetroxyla, Bimeda, Ireland) and buprenorphine hydrochloride (0.3 ml/kg body weight, Hypnorm, Janssen Pharmaceutical, Belgium), supplemented with diazepam (5 mg/ml, 1 mg/kg body weight, Valium 10, Roche). In order to maintain the level of neuroleptanalgesia, increments of Hypnorm (0.1 ml/kg) were given at 30-minute intervals during the operation.

The surgical procedure consisted of the creation of two 10 × 5 mm^2 ^full-thickness (approximately 2 mm) cranial defects, devoid of periosteum, using templates, in the parietal bones. The defects were produced with round stainless steel burs (1 mm in diameter) on a low speed dental drill. Outlines of the defects were made initially by making holes of full thickness in the parietal bone with a stainless steel wire template bent to the required size of the defect. The holes were joined to complete the process. During the cutting of the bones, copious amounts of sterile saline were used for irrigation and to minimize thermal damage to the tissues. In the test animal, the defects were filled with 0.02 g of purified absorbable fibrillar collagen matrix (Collagen Matrix, USA) with 0.2 ml of RYR extract (1 g/ml). The grafts were prepared 15 minutes before grafting. In the control animal, the defects were grafted with 0.02 g of the same collagen matrix with 0.2 ml water for injection.

All wounds were closed with interrupted 3/0 black silk sutures. No attempt was made to approximate the periosteum to prevent the barrier effect. Postoperatively, the rabbits were given oxytetracycline hydrochloride daily for ten days and buprenorphine hydrochloride daily for two weeks.

Two weeks after surgery, the animals were sacrificed with sodium pentobarbitone. Immediately after death, defects and surrounding tissues were removed for histological preparation and examination.

Tissues were fixed in 10% buffered formal saline solution, demineralized with K's Decal Fluid (sodium formate/formic acid) and double embedded in celloidin-paraffin wax. Serial, 5-μm-thick sections of the whole defect were cut perpendicular to the long axis. The slides were stained with Periodic acid-Schiff stain for identification of new bone formation.

### In vitro quantitative study

#### Cell culture

UMR 106, a rat osteoblastic cell line derived from osteosarcoma, was used as a model system for *in vitro *studies of the effect of parathyroid hormone on osteoblasts [11]. UMR 106 cells (ATCC, CRL-1661) were cultured in Dulbecco's modified Eagle's medium (Gibco, UK) which contained fetal bovine serum (10% vol, Gibco, UK) and antibiotics penicillin G sodium (100 units/ml, Gibco, UK) and streptomycin (100 μl/ml, Gibco, UK), and incubated at 37°C in a humidified atmosphere of 5% CO_2 _and 95% air [12].

In the control group, cells were cultured without intervention for three durations (24 hours, 48 hours and 72 hours). In the RYR group, cells were cultured with RYR extract of various concentrations (0.001 g/ml, 0.005 g/ml and 0.01 g/ml) for three durations (24 hours, 48 hours and 72 hours) in 96-well tissue culture plates (4–5 × 10^4 ^cells/well, Corning, USA). Each experiment had eight samples and was repeated once.

#### BCA assay for total protein

The total protein is an indicator for the biosynthetic capacity of bone cell cultures. The bicinchoninic acid (BCA) assay is a biochemical assay for determining the total level of protein in a solution using colorimetric techniques [13]. This method combines the reduction of the Cu^2+ ^to Cu^+ ^by proteins in an alkaline medium with the highly sensitive and selective colorimetric detection of the Cu^+ ^using a BCA containing reagent [14]. The purple-colored reaction product of this assay is a chelation of two BCA molecules with one Cu^+^. This water-soluble compound exhibits a strong absorbance at 562 nm and is linearly proportional to the protein concentrations over a broad range (20–2000 μg/ml) [15]. Cells were lysed and the cellular material was transferred to 250 μl of buffer (10 mM Tris HCl, pH 7.5, 0.5 mM MgCl_2 _and 0.1% Triton-X100). The cellular material was homogenized by two freeze-and-thaw cycles [15]. The cellular protein concentration was determined by a BCA protein assay kit (Pierce, USA).

#### MTT assay for cell viability and mitochondrial activity

The cell viability and mitochondrial activity of the bone cells after exposure to various concentrations of RYR extract were determined by the colorimetric MTT assay which detects the conversion of 3-(4,5-dimethylthiazol-2-yl)-2,5-diphenyltetrazolium bromide (MTT, Sigma, USA) to a purple formazan product. MTT is a pale yellow tetrazolium salt that produces a dark blue formazan product when incubated with living cells. MTT ring is cleaved in active mitochondria in living cells [13]. The MTT assay was used to measure the bone cell viability and mitochondrial activities. Cells were incubated with 0.5 mg/ml of MTT in the final four hours of each interval. The medium was then decanted; the formazan salt was dissolved in 150 μl of acid-isopropanol; and the optical density was determined at 570 nm against a reference wavelength of 690 nm with an ELISA reader [13].

#### ALP assay for osteogenic activity

Alkaline phosphatase (ALP) assay is a standard method to measure osteogenic activity of bone cells *in vitro*. The ALP assay protocol of Declercq *et al*. [12] was followed. Mono-layers of cultured cells were rinsed with Ringer solution. Cells were lysed and the cellular material was transferred to 250 μl of buffer (10 mM Tris HCl, pH 7.5, 0.5 mM MgCl_2 _and 0.1% Triton X-100). The cellular material was homogenized by two freeze-and-thaw cycles. The ALP assay was performed using p-nitrophenylphosphate as substrate. Each sample (50 μl) was added to 50 μl of p-nitrophenylphosphate (4.34 mM) in buffer (100 mM glycine, pH 10.3, 1 mM MgCl_2_). The mixture was incubated at 37°C for 30 minutes on a bench shaker. The enzymatic reaction was stopped by adding 50 μl of 1 M NaOH. The reaction product was quantified by absorbance measurement at 405 nm against p-nitrophenol (PNP) standards. The ALP activity is expressed as mM PNP/μg protein.

#### Statistical analysis

Data were analyzed with software SPSS 15.0 for Windows (SPSS, USA). Normality test (Kolmogorov-Smirnov test, P > 0.05) was performed. The unpaired t-tests with Bonferroni correction (P = 0.05) were used to compare the control with various concentrations of RYR extract (0.001 mm, 0.005 mm and 0.01 mm). The results with P < 0.05 were considered to be statistically significant.

## Results

### In vivo qualitative study

All animals remained in excellent health throughout the course of the experiment and recovered rapidly after operation. There was no evidence of side effects or infection in any of the animals.

**Figure 2:**
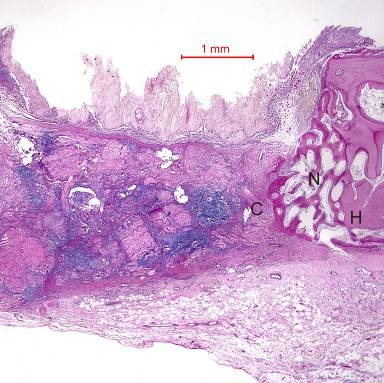
**Photomicrograph of bone defect grafted collagen matrix mixed with RYR extract (test) on day 14.** New bone (N) was observed across the defect. H = Host bone. Some collagen matrix (C) remained at the center of the bony defect (Periodic acid-Schiff stain, original magnification ×40).

In the rabbit grafted with RYR extract in collagen matrix, new bone was formed at the host bone/graft interface tended to grow across the defect (Figure 2). Integration of the RYR extract and collagen with the recipient bed was characterized by the appearance of new bone. No cartilage was found. At higher magnification, new bone could be seen growing around a collagen matrix fragment and tended to growth towards and amalgamate with the collagen matrix. The presence of bone cells indicated that new bone was formed.

**Figure 3:**
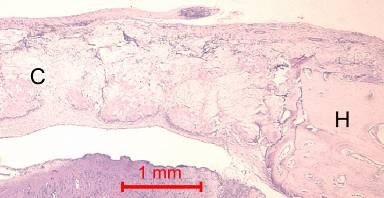
**Photomicrograph of bone defect grafted with collagen matrix alone (control) on day 14.** No new bone was observed across the defect. There was new bone near the ends of the host bone (H). Collagen matrix (C) remained across the bone defect (Periodic acid-Schiff stain, original magnification ×40).

In the control animal, little new bone was formed at the host bone/graft interface. Some collagen fibers were present at the center of the defects (Figure 3). In both rabbits, the defect was healed, with fibrous tissue bridging across the defect.

### In vitro quantitative study

#### BCA assay

RYR extract at all the tested concentrations did not show any statistically significant effect on total protein.

#### MTT assay

RYR extract increased the optical density of the purple formazan product in the MTT assay. The effect was most consistent at 48 hours where the increase ranged from 60% to 74%. At 24 hours, only RYR extracts of 0.001 g/ml and 0.005 g/ml showed statistically significant effects. At 72 hours, only RYR extract of 0.001 g/ml showed a statistically significant effect. RYR extract of 0.01 g/ml showed a statistical significant effect at 24 hours but not at 72 hours (Table 1).

**Table 1:** MTT assay of UMR 106 cells

Concentration of RYR extract (g/ml)	Control	0.001	0.005	0.01
Optical density (0 hour) mean (SD)	0.196 (0.019)	-	-	-
Optical density (24 hours) mean (SD)	0.218 (0.052)	0.287 (0.018)	0.342 (0.033)	0.257 (0.040)
P-value*		0.021	< 0.001	NS
Optical density (48 hours) mean (SD)	0.177 (0.031)	0.282 (0.048)	0.293 (0.014)	0.306 (0.044)
P-value*		< 0.001	< 0.001	< 0.001
Optical density (72 hours) mean (SD)	0.360 (0.049)	0.421 (0.035)	0.360 (0.014)	0.372 (0.031)
P-value*		0.036	NS	NS

* Unpaired t-tests with Bonferroni correction were performed to compare with the control of various RYR extract concentrations.

Optical density: optical density of the purple formazan product at 570 nm

NS: not statistically significant

#### ALP assay

RYR extract increased ALP activity in all groups. RYR extract of 0.005 g/ml significantly increased ALP activity at 48 hours and 72 hours. RYR extract of 0.001 g/ml significantly increased ALP activity only at 48 hours. RYR extract of 0.01 g/ml significant increased ALP activity ranging from 16% to 31% in all three time durations (Table 2).

**Table 2:** ALP assay of UMR106 cells

Concentration of RYR extract (g/ml)	Control	0.001	0.005	0.01
PNP (0 hour) mean (SD)	8.26 (0.95)	-	-	-
PNP (24 hours) mean (SD)	8.14 (0.53)	7.50 (2.39)	7.07 (1.54)	9.74 (1.37)
P-value*		NS	NS	0.039
PNP (48 hours) mean (SD)	5.58 (0.57)	6.62 (0.84)	6.40 (0.50)	6.46 (0.64)
P-value*		0.033	0.024	0.033
PNP (72 hours) mean (SD)	4.50 (0.32)	3.89 (0.80)	5.17 (0.42)	5.81 (0.87)
P-value*		NS	0.010	0.012

* Unpaired t-tests with Bonferroni correction were performed to compare the control with samples of various RYR extract concentrations.

PNP: mM p-nitrophenol (PNP) per μg protein of RYR extract

NS: Not statistically significant

## Discussion

In comparison with the control, treatment of bone defects with RYR extract was demonstrated *in vivo *to have enhanced new bone formation. Treatment of UMR 106 osteosarcoma cells with RYR extract was found to have increased cell viability and mitochondrial activity as measured by the MTT assay and higher osteogenic activity as detected by the ALP assay. The effect of RYR extract on new bone formation might not be non-specific (*e.g*. general biosynthetic activities in the cells) because no increased total protein was detected. It is to be confirmed whether the statin-like substances in RYR extract are the main osteogenic agents in RYR extract. The specific mechanism of the RYR osteogenic agents is also to be investigated.

A previous study indicated a temporal expression pattern of different markers during the osteoblast development [16]. Different markers (*e.g*. ALP and osteopontin) showed an increase and then decrease in expression during different stages. It is possible that the MTT response varies during the osteoblast development. As UMR 106 cells express ALP in their basal state [17], only potent osteogenic agents can significantly increase ALP activity in UMR 106 cells.

The results suggest that the effect of RYR extract may be dose-dependent and that a drop of the ALP activity at 48 hours and 72 hours may be a result of an increase in cellular proliferation offsetting an increase in ALP activity.

RYR extract contains several statin-like components that inhibit the HMG-CoA reductase. Statin-mediated activation of BMP-2 promoter was inhibited by mevalonate which is the downstream metabolite of HMG-CoA reductase [5]. The effect of RYR extract on bone formation suggests that the inhibition of HMG-CoA reductase in the mevalonate pathway may increase bone cells. Further studies on this pathway and its relation to bone formation may help reveal some of the RYR's biochemical actions and potential uses in treating bone defects and osteoporosis.

## Conclusion

RYR extract stimulated new bone formation in bone defects *in vivo *and significantly increased bone cell formation *in vitro*. RYR is a natural product with potential in treating bone defects and probably also osteoporosis.

## Competing interests

The author(s) declare that they have no competing interests.

## Authors' contributions

RW conceived and designed the study. RW and BR conducted the actual research work including supervision of technicians. Both RW and BR contributed to the paper writing. Both authors read and approved the final version of the manuscript.
